# Formes hémorragiques de Dengue observées dans le service des maladies infectieuses du CHU Yalgado Ouédraogo, Burkina Faso

**DOI:** 10.11604/pamj.2016.23.168.9234

**Published:** 2016-04-07

**Authors:** Savadogo Mamoudou, Boushab Mohamed Boushab

**Affiliations:** 1 Service des Maladies Infectieuses du CHU YO, Burkina Faso; 2 Service de Médecine Interne du Centre Hospitalier d'Aïoun, Mauritanie

**Keywords:** Dengue hémorragique, arbovirose, Burkina Faso, Hemorrhagic dengue, arbovirus, Burkina Faso

## Abstract

La dengue est une arbovirose ré émergente dans les pays tropicaux. Sa forme hémorragique peut s'accompagner d'une défaillance circulatoire pouvant conduire à un choc hypovolémique, souvent fatal. Nous rapportons une série de trois cas de formes hémorragiques de dengue observées dans le service des maladies infectieuses du CHU Yalgado Ouédraogo de Ouagadougou dont l'objectif est de décrire les caractéristiques épidémiologiques cliniques et évolutives. Les cas étaient des jeunes de sexe féminin dont l’âge variait entre 27 ans et 43 ans, résidant dans la ville de Ouagadougou. Malgré de multiples transfusions de concentrées plaquettaires et un traitement symptomatique, nous déplorons un cas de décès. Le développement d'antiviraux et la mise au point de nouveaux vaccins constituent une lueur d'espoir pour prévenir la létalité due à cette maladie.

## Abstract (en)

Dengue is a re-emerging arboviral disease in tropical countries. Haemorrhagic form may be associated with circulatory failure and produce hypovolemic shock, which is often fatal. We report a series of three cases of hemorrhagic dengue observed at the Infectious Diseases Department CHU Yalgado Ouédraogo, Ouagadougou, in order to describe its epidemiological, clinical, and evolutive characteristics. Our study included young females who had an onset age of between 35 years and 45 years, residing in the city of Ouagadougou. Despite multiple concentrated platelets transfusions and symptomatic treatment, we mourned a death. The development of antivirals and of new vaccines is a silver lining to prevent dengue lethality.

## Introduction

La dengue est une infection virale qui touche entre 100 et 400 millions de personnes dans le monde [1], pour l'essentiel dans les régions tropicales et subtropicales [2, 3]. Elle est transmise par les piqures des moustiques (*Aedes aegypti ou Aedes albopictus*) [3, 4]. C'est une arbovirose ré émergente endémique dans plus de 100 pays, avec une prédilection pour les zones urbaines et péri-urbaines. Répandue dans le monde, c'est l'une des premières causes des fièvres hémorragiques virales [3]. Selon les estimations de l'organisation mondiale de la santé (OMS), environ 20 000 personnes décèdent chaque année [5]. Le nombre annuel de cas de dengue est en augmentation dans le monde [4–6]. Cette augmentation est probablement liée à l'hyper-endémicité des quatre sérotypes du virus de la dengue. Les symptômes sont généralement bénins, mais dans les cas graves, des hémorragies peuvent survenir entrainant parfois la mort dans 5% des cas [4, 7]. Dans les formes hémorragiques, une fièvre, des hémorragies, une hépatomégalie peuvent être observée et dans les cas les plus sévères, des signes de défaillance circulatoire avec fuite plasmatique pouvant conduire à un choc hypovolémique [4, 7]. Nous rapportons trois cas de dengue hémorragique observés dans le service des maladies infectieuses du CHU Yalgado Ouédraogo de Ouagadougou entre 2013 et 2015.

## Patient et observation

### Cas 1

Patiente de 27 ans sans antécédent pathologique particulier est admise au service des urgences du Centre hospitalier universitaire Yalgado Ouédraogo le 7/11/ 2013 pour hyperthermie, obnubilation et vomissement. La symptomatologie remonterait à deux semaines environ marquée par la survenue d'un syndrome grippal fait de fièvre, céphalées, dysphagie et arthralgies. Une goutte épaisse (GE) et un test de diagnostic de la dengue sont revenus positifs. L'hémogramme montrait une hyperleucocytose à 11 900/mm3 et une thrombopénie à 42 000/mm3. Un traitement à base d'arthémeter fut alors institué. L’évolution de la maladie a été marquée par l'exacerbation des signes accompagnés d'hématémèses d'où son transfert au service des Maladies Infectieuses de l'Hôpital Yalgado Ouédraogo. L'examen clinique à l'entrée notait un état général conservé, des conjonctives bien colorées anictériques, une température à 37,8°C, une TA à 130/70 mmHg, un pouls à 102/mn. L'examen de la peau et des phanères notait en regard du mollet droit et de l’épaule gauche, des tâches ecchymotiques et un hématome au niveau de l'aine droite, point de prélèvement sanguin. Le reste de l'examen clinique était sans particularité. Le diagnostic retenu était une dengue hémorragique associée à un paludisme grave. Le traitement a consisté en une transfusion de concentré de plaquette et du sang total. Cette transfusion a été associée à une antibiothérapie (ofloxacine), un traitement antipaludique (arthémeter) et un traitement antalgique (perfalgan). L’évolution été favorable sous ce traitement et le patient est sorti de l'hôpital au bout de six jours hospitalisation.

### Cas 2

Patiente de 35 ans a été admise le 26/10/2015 pour douleur au flanc droit associé à une hématurie. La symptomatologie remonterait à cinq jours environ marqué par l'apparition d'une colique néphrétique associée à des brulures mictionnelles, à une hématurie totale et à des vomissements. Cette symptomatologie l'avait amenée consulter dans une formation sanitaire privée de la ville de Ouagadougou d'où elle est référée aux urgences médicales du CHU Yalgado Ouédraogo pour meilleure prise en charge. L'examen à son admission notait une conscience claire, des conjonctives moyennement colorées anictériques, une température à 39^°^C, une fréquence respiratoire à 21 cycles/mn, une fréquence cardiaque à 102 bat/mn, une tension artérielle à 110/80 mmHg. L'examen de l'appareil urinaire notait des urines hématiques, une douleur à la palpation des points urétéraux supérieurs et moyens, il n'avait pas de voussure hypogastrique. L'examen de la peau et des phanères notait des tâches ecchymotiques au niveau du membre supérieur droit et au niveau du pli du coude homolatéral. Il y avait également des tâches purpuriques au niveau de la cuisse gauche. L'examen biologique montrait une hyperleucocytose à 14 300/mm3, une anémie à 8g/dl, une thrombopénie sévère à 61 000 à l'admission, la créatininémie à 65 miromol/l, la glycémie à 5,49 mml/l, le taux de prothrombine (TP) à 61,3%. La sérologie de la dengue faite le 30/09/2015 montrait une absence de l'antigène NS1, une absence de l'anticorps anti NS1 de type IgM, et la présence de l'anticorps NS1 type IgG. L’échographie abdominale objectivait la présence de caillot dans la vessie, l'examen des selles à la recherche des kystes œufs et parasites(KOP) montrait la présence de *Trichomonas intestinalis*, et l'examen parasitologique des urines montrait une absence d’œuf de *Shistosoma haematobium*. Le traitement institué était à base de concentrée plaquettaire en raison d'une poche/10kg de poids corporel, du sang total, des perfusions de solutés de remplissage, une antibiothérapie à base de Bectacef + flagyl, du perfalgan. L’évolution a été favorable et elle est sortie de l'hôpital le 21/11/2015.

### Cas 3

Patiente de 43 ans ayant des antécédents de toxémie gravidique compliquée de d'insuffisance rénale et d'hypertension artérielle (HTA) suivie en néphrologie depuis 2005 et sous metyl-dopa (Aldomet^®^), a été admise dans le service des maladies infectieuses le 11/11/2015 pour céphalées asthénie vertiges vomissement hémoptysie et de fièvre. La patiente était dans une formation sanitaire privée où elle aurait reçu des soins à base d'arthémeter + lumefantrine. Vue la persistance de la symptomatologie, une sérologie de la dengue est demandée le 12/11/2015 et les résultats montraient les AgNS1 positif, les Ac IgM et Ig G anti NS1 négatifs. A la numération formule sanguine, les globules blancs étaient revenus à 3 190/mm3, les plaquettes à 125 000/mm^3^, le taux d'hémoglobine à 13g/dl, la créatininémie à 86 µmol/l, la glycémie à 15,03 mmol/l, la CRP à 44,18mg/l. Elle est référée au CHU YO pour meilleure prise en charge. L’évolution a été marquée le 19/11/2015 par une altération de l’état général avec une obnubilation de la conscience, une agitation, un délire, une oligurie, une hématémèse, la température à 37^°^7, la fréquence cardiaque à 92/mn, la fréquence respiratoire à 24cycles/mn, le pouls à100/mn. L'examen biologique du 16/11/2015 montrait une créatininémie à 268,4 µmol/l et celui du 19/11/2015 à 813,2 µmol/l, ayant nécessité de séance de dialyse. Quant aux plaquettes leur taux a évolué de 89000/mm^3^ le 16/11/2015 à 24000/mm^3^ le 17/11/2015, puis 29 000/mm^3^ le19/11/2015. En plus de la dialyse, le traitement institué était fait de transfusion de concentrées plaquettaires, de perfusion de solutés glucosé isotonique avant son transfert dans un service de réanimation polyvalente où il décédait le 20/11/2015 dans un tableau d'insuffisance rénale aigue oligurique associée à des métrorragies et des hématémèses.

## Discussion

La dengue est la première cause de fièvre hémorragique virale dans le monde [3]. Sur le plan clinique, l'infection peut être asymptomatique mais lorsqu'elle est symptomatique ses manifestations sont assez polymorphes. L'OMS distingue la dengue classique qui est a priori bénigne, de la dengue hémorragique qui est grave [1, 8]. Bien que des cas d'hépatites graves, d'encéphalites ou d'infections opportunistes soient fréquemment rapportés à l'origine des cas mortels de dengue, la classification de l'OMS sur la sévérité des cas de dengue ne les prend pas en compte [3]. Seule la physiopathologie de la dengue hémorragique a fait l'objet de beaucoup de spéculations. Plusieurs hypothèses sont émises dont celles de l’*Antibody Dependent Enhancement* (ADE) ou augmentation de l'infection dépendante des anticorps. Cette hypothèse soutient que la dengue hémorragique surviendrait essentiellement au cours une infection secondaire. Les mécanismes à l'origine de la fuite plasmatique ne sont pas connus; ils mettraient en jeu des altérations transitoires des endothéliums vasculaires [3, 5, 9]. Mais la survenue avérée de choc de dengue lors d'infections primaires ne peut pas être expliquée par l'hypothèse de l'ADE [3, 9, 10]. En ce qui concerne le diagnostic de la dengue hémorragique, plusieurs tests sont disponibles comme la détection de l'ARN viral par la PCR et les examens sérologiques. Devant un cas suspect de dengue, la positivité de l'AgNS1 et des IgM permet de faire un diagnostic précoce. Alors que lors d'une infection secondaire, ce sont les IgG qui apparaissent plus précocement [3].

## Conclusion

La dengue est une arbovirose ré émergente. Sa forme hémorragique nécessite une unité de soins intensifs avec souvent de multiples transfusions de concentrées plaquettaires. Le développement d'antiviraux et la mise au point de nouveaux vaccins constituent un espoir pour la réduction de la létalité due à cette arbovirose. Le renforcement des plateaux techniques des laboratoires dans notre contexte burkinabè est nécessaire pour une bonne surveillance épidémiologique de la maladie.
